# Correlates of Restless Legs Syndrome in Older People

**DOI:** 10.3390/jcm13051364

**Published:** 2024-02-28

**Authors:** Magdalena Szklarek, Tomasz Kostka, Joanna Kostka

**Affiliations:** 1 Department of Geriatrics, Medical University of Lodz, 90-647 Lodz, Poland; magdalena.szklarek@gmail.com (M.S.); tomasz.kostka@umed.lodz.pl (T.K.); 2 Department of Gerontology, Medical University of Lodz, 93-113 Lodz, Poland

**Keywords:** older adults, comprehensive geriatric assessment, alcohol consumption, number of medications, quality of life

## Abstract

**Background**: We examined the association between restless legs syndrome (RLS) and comprehensive geriatric assessment (CGA) data in two older European populations. The second goal was to evaluate correlates of their quality of life (QoL). **Methods**: Diagnostic criteria of the International RLS Study Group (IRLSSG) and elements of CGA were used in this study. **Results**: Among the examined 246 participants, 77 (31.3%) suffered from RLS, more often in the UK (39.4%) than in Poland (25.4%) (*p* = 0.019). In the multivariate logistic regression model, female sex [OR (CI) = 3.29 (1.51–7.21); *p* = 0.0014], the number of medications per day [OR (CI) = 1.11 (1.02–1.20); *p* = 0.011] and alcohol consumption [OR (CI) = 5.41 (2.67–10.95); *p* < 0.001] increased the probability of RLS. Residing in Poland [OR (CI) = 3.06 (1.36–6.88); *p* = 0.005], the presence of RLS [OR (CI) = 2.90 (1.36–6.17); *p* = 0.004], chronic heart failure, [OR (CI) = 3.60 (1.75–7.41); *p* < 0.001], osteoarthritis [OR (CI) = 2.85 (1.47–5.49); *p* = 0.0016], and urinary incontinence [OR (CI) = 4.74 (1.87–11.9); *p* < 0.001] were associated with a higher probability of mobility dimension problems in the QoL. Higher physical activity was related to a lower probability of mobility problems [OR (CI) = 0.85 (0.78–0.92); *p* < 0.001]. **Conclusions**: female sex, the number of medications and alcohol consumption are independent correlates of RLS in older adults. RLS together with several chronic medical conditions and a low physical activity level were independent correlates of the mobility dimension of the QoL.

## 1. Introduction

Restless legs syndrome (RLS) is associated with unpleasant sensory experiences, with usual onsets in the evenings and nights. It occurs in the extremities and in extreme cases, it can also involve the torso, making rest difficult during a sleepless night. This sensory-motor neurologic disorder often dramatically affects sleep and QoL [1]. The lifetime prevalence of RLS is estimated to be within 2% to 3% of the adult population [2]. Since the very first description noted by Oxford physician Thomas Willis in the XVII century, RLS, also known as Willis–Ekbom disease, has been described in well over 5000 articles published on PubMed [3].

The pathophysiology of RLS is only partially understood [4,5]. The prevalence of RLS is higher in older people and females. RLS is most commonly related to iron deficiency and iron-deficiency anemia, pregnancy, uremia and polyneuropathies [6,7]. Brain iron deficiency, toxic metal exposure, concomitant diseases and postinfectious immunological mechanisms may influence the production of RLS symptoms [8,9,10,11,12]. Of individuals with conditions associated with iron-deficiency states, including pregnancy, renal failure, and anemia, 25–30% of them may develop RLS [13]. Patients with RLS could be deficient in vitamins D and B12 [14,15]. Various comorbidities such as kidney disease, cardiovascular diseases, diabetes mellitus, hypothyroidism, chronic liver disease, and neurological, rheumatological and respiratory disorders accompany RLS [2,16,17,18,19]. In our recent study, a significantly higher number of amalgam dental fillings were found in older adults with RLS as compared to the subjects without RLS symptoms [20].

As the prevalence of RLS increases with advancing age substantially decreasing the QoL of older adults, this population should be among the primary targets of research on RLS epidemiology and pathophysiology [21]. Several lifestyle factors have been linked to the occurrence of RLS in the general adult population [22]. Nevertheless, limited data exist on factors predisposing to RLS occurrence in older adults. Specfically, there are no studies relating RLS to comprehensive geriatric assessment (CGA) data and assessing the potential contribution of RLS to the quality of life (QoL) of older adults taking into account multiple covariates of CGA. Our study aimed to examine the association between RLS and an extensive set of CGA-potentially related factors such as smoking, alcohol consumption, concomitant diseases, physical and cognitive functioning, medications used, nutritional status and physical activity, in two older European populations. The second goal of the study was to evaluate correlates of the QoL of those subjects.

## 2. Materials and Methods

### 2.1. Subjects

The study population comprised 246 subjects (63 males and 183 females, median age 79 years) who volunteered to participate in the study, and was composed of two groups. The first was 104 subjects living in a Polish Housing Society in Penrhos, North Wales, founded in 1949, providing accommodation and support to Polish ex-service men and women who remained in the UK following World War II. The second group was 142 outpatients of the Geriatric Clinic of the Medical University of Lodz, Poland.

The exclusion criteria were having a known anemia, chronic kidney disease, known iron or vitamin deficiency, terminal illness, major disabilities or severe dementia. The criteria for inclusion in the study were being of an age > 60 years, having satisfactory verbal communication, the ability to perform functional tests and give written consent to participate in the study.

The study was approved by the Bioethics Committee of the Medical University of Lodz and complies with the Declaration of Helsinki and Good Clinical Practice Guidelines.

### 2.2. Methods

All subjects were interviewed to obtain a full medical history including regular medication taken. The medical history was supplemented based on the patient’s medical records. In case of doubt, the interview was further supplemented by a conversation with a nurse or caregiver. Information about alcohol intake and smoking was gathered. Alcohol consumption was classified as “yes” if any amount of alcoholic beverages were consumed during the last 7 days. Current smoking was defined as smoking at least part or all of a cigarette during the past 30 days [23]. Arm blood pressure was measured once.

The diagnosis of RLS is purely clinical and is based on the information obtained during an interview with the patient. We applied the diagnostic criteria of the International Restless Legs Syndrome Study Group (IRLSSG) in the form of four questions from an internationally used questionnaire in order to determine the appearance of the problem of RLS [24].

The analysis of factors coexisting with RLS based on a comprehensive geriatric assessment (CGA) was conducted. A functional efficiency assessment was made using the ADL (Activities of Daily Living) scale [25] and the instrumental functioning scale—IADL (Instrumental Activities of Daily Living) [26]. The ADL scale is used to assess the basic activities of daily living, such as bathing, use of the toilet, continence, dressing, eating and mobility. One point (max 6 points in total) is awarded for the ability to perform a given activity. The IADL scale is a tool used to assess the ability to live independently in the community. It contains 8 questions (max 8 points in total) about complex daily activities regarding using the phone, shopping, food preparation, cleaning, washing, using means of transport, using medication and using money.

The TUG test is a popular test used to assess functional ability. It involves performing a series of activities over time (time is measured with a stopwatch): getting up from a chair, walking for a distance of 3 m, turning around, walking again for a distance of 3 m and sitting on the chair. A result above 14 s is considered to be at an increased risk of falling [27].

Physical activity assessments were conducted using two questionnaires: the seven-day recall PA questionnaire [28] and the Stanford questionnaire [29]. The seven-day recall PA questionnaire is designed to determine the average daily physical activity energy expenditure (PA-EE) over the past 7 days (kcal·kg^−1^ day^−1^). Energy expenditure is calculated based on time spent sleeping and the light, moderate, vigorous and very hard activities over the past week. The Stanford Moderate Index was used to assess health behaviors related to physical activity (PA-HRB). This index is determined on the basis of 6 questions about habitual behaviors of light and moderate intensity. One point is awarded for each confirming answer (maximum 6 points in total).

The nutritional status of the study group was assessed using tools such as the MNA (Mini Nutritional Assessment) questionnaire [30], and anthropometric measures, including body mass and height (RADWAG personal weight scales, Radom, Poland) as well as waist and calf circumferences (SECA measuring tape, Hamburg, Germany). The body mass index (BMI) was calculated by dividing body weight by height in meters squared. The MNA is a questionnaire recommended for assessing the nutritional status of older people. It consists of parts such as a general assessment, anthropometric measures, dietary assessment and self-assessment. The full version was used in the study, consisting of 18 questions regarding various aspects of the risk of malnutrition. In the test, it is possible to obtain from 0 to 30 points, with a score of 24 points and above indicating a normal level of nutrition, 17–23.5 points indicating a risk of malnutrition, and a score below 17 points indicating malnutrition.

Mental state was assessed using the GDS (Geriatric Depression Scale) [31] and a short mental state assessment scale—the MMSE (mini-mental state examination) [32]. The short form of the GDS is a tool used to screen and assess symptoms of depression in older people. The scale consists of 15 questions related to mood disorders with possible answers of “yes” or “no”. The GDS-15 is scored as follows: 0 to 5 points—normal, 6–10 points—a risk of depression and 11 points or more indicates depression. The MMSE is a clinical scale used to examine disorders in a patient’s cognitive functioning. The scale assesses areas of mental abilities, including their orientation in time and place, attention/concentration, short-term memory, language skills, visuospatial abilities, and their ability to understand and follow instructions. During the test, it is possible to obtain a maximum of 30 points, where 27 to 30 points is normal, 24 to 26 points indicates mild cognitive impairment without dementia, 19 to 23 points—mild dementia, 11 to 18—moderate dementia and less than 11 suggests severe dementia.

The QoL was assessed by the international EQ-5D questionnaire [33]. The questionnaire consists of two parts. The first one assesses the existence of a problem in five domains of functioning (mobility, self-care, usual activities, pain/discomfort, and anxiety/depression). Each domain is assessed on a 3-point scale (1—no problem, 2—some problems, 3—severe problems). In the second part, using a 100-point visual analog scale (VAS), participants determine their perception of their overall health. Zero on this scale means the worst imaginable health status, while 100 means excellent health.

### 2.3. Statistical Analysis

The results were verified for their normality of distribution and equality of variance. The one-way analysis of variance (ANOVA), Mann–Whitney test or chi-square test were used to compare the groups. The EQ-5D dimension data were dichotomized (no problems vs. any problem) for statistical analyses. A multiple logistic regression was used to select independent correlates of RLS or QoL data with independent variables significant in bivariate associations entering the regression models. A multivariate logistic regression model was constructed by employing the forward–backward stepwise selection procedure. Odds ratios (OR) and confidence intervals (CI) with 95% confidence limits were calculated. The quantitative variables are presented as the mean ± standard deviation or median and interquartile range [25–75%], qualitative variables as numbers and percentages. The statistical analysis was performed using Statistica (version 13.3) software (StatSoft, Kraków, Poland). The limit of significance was set at *p* = 0.05 for all analyses.

## 3. Results

### 3.1. Demographic and Clinical Data

Among the examined 246 participants, 183 (74.4%) were women. The proportion of men was smaller in Poland (19%) than in the UK (34.6%), *p* < 0.001. The English group was significantly older, *p* < 0.001 (the mean age in the UK was 83.5 ± 7.6 and in Poland was 75.8 ± 8.4 years). Seventy-seven (31.3%) subjects suffered from RLS, significantly more often in the UK (39.4%) than in Poland (25.4%) (*p* = 0.019). People from the Polish group smoked more cigarettes per day but consumed less alcohol (both *p* < 0.001).

Among the 246 participants, 71.1% were diagnosed with arterial hypertension, 37.4% had hypercholesterolemia, 22.8% suffered from diabetes mellitus, 39.4% had ischemic heart disease, 49.6% had chronic heart failure, 13.8% of the individuals had a myocardial infarction while 15.4% had a stroke in the past. The Polish group suffered more often from arterial hypertension (*p* = 0.001), ischemic heart disease and chronic heart failure (both *p* < 0.001), and stroke (*p* = 0.030). The English group suffered more often from hypercholesterolemia (*p* = 0.003) and ophthalmologic diseases (*p* = 0.012). The number of infections per year was higher in the Polish group and this group had fewer influenza vaccinations per year (both *p* < 0.001). The English group was taking more medications per day than the Polish (*p* = 0.005) and had higher systolic blood pressure (*p* < 0.001). English participants had higher ADL and IADL, MNA screening and EQ-5D VAS scores, but lower GDS and EQ5D dimensions scores (*p* < 0.001). The English group had a lower total energy expenditure (kcal/kg/day) (*p* < 0.001) but higher score for the Stanford Moderate Index (*p* = 0.041).

**Figure 1:**
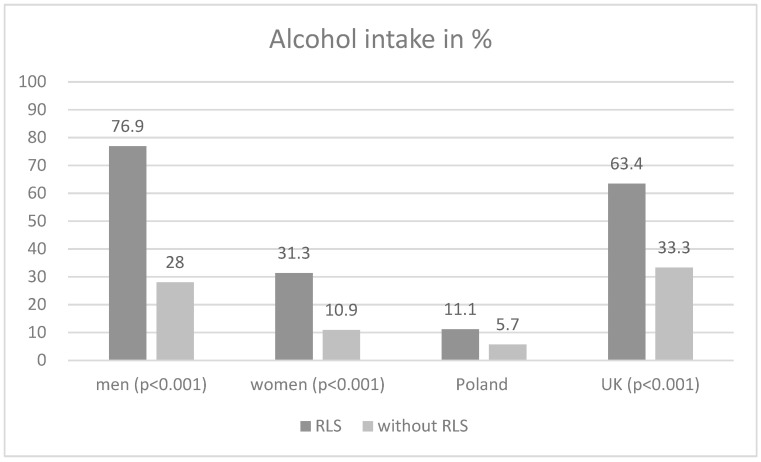
Prevalence of alcohol consumption in relation to the presence of RLS according to sex and country.

Table 1 shows the characteristics of the whole studied population divided into two groups, with and without RLS. Both groups had a similar age and smoking status. RLS was more frequent in women than in men. The consumption of alcohol was significantly higher in the group with symptoms of RLS (39.0%) than in those without RLS (16.0%), which was similar when analyzed separately in women and men (*p* < 0.001). The prevalence of alcohol consumption in relation to the presence of RLS according to sex and country is shown in Figure 1. The prevalence of concomitant diseases was not different between the RLS and non-RLS groups. The number of medications used per day and IADL scores (borderline significance) were higher in the RLS group. Other measures of the comprehensive geriatric assessment were not statistically different between the RLS and non-RLS groups.

Table 2 shows the QoL indices in the groups with and without RLS. The prevalence of mobility problems was higher in the RLS group, other indices were not statistically different.

### 3.2. Multivariate Regression Models

The age, sex, country of participants and all variables significantly related to RLS in bivariate associations entered the multivariate logistic regression model. The results are presented in Table 3. In the stepwise selection procedure, sex, the number of medications per day and alcohol consumption were identified as independent correlates of RLS. Female sex [OR (CI) = 3.29 (1.51–7.21); *p* = 0.0014], the number of medications per day [OR (CI) = 1.11 (1.02–1.20); *p* = 0.011] and alcohol consumption [OR (CI) = 5.41 (2.67–10.95); *p* < 0.001] increased the probability of RLS. Forward and backward stepwise regression models gave the same results.

Age, physical activity (kcal/kg/day), RLS, and the presence of chronic heart failure, osteoarthritis and urinary incontinence were related to mobility dimension problems of the EQ-5D questionnaire. These variables were included in the multivariate regression models together with sex and country of residence. Although related to the mobility dimension, TUG and ADL results were not included in the multivariate models because of their obvious overlapping characteristics to mobility. Country of residence, RLS, the presence of chronic heart failure, osteoarthritis and urinary incontinence as well as physical activity level (kcal/kg/day) were selected as independent correlates of mobility dimension problems. Residing in Poland was related to a higher probability of mobility problems [OR (CI) = 3.06 (1.36–6.88); *p* = 0.005]. The presence of RLS [OR (CI) = 2.90 (1.36–6.17); *p* = 0.004], chronic heart failure, [OR (CI) = 3.60 (1.75–7.41); *p* < 0.001], osteoarthritis [OR (CI) = 2.85 (1.47–5.49); *p* = 0.0016], and urinary incontinence [OR (CI) = 4.74 (1.87–11.9); *p* < 0.001] were associated with a higher probability of mobility problems. Higher physical activity was related to a lower probability of mobility problems—a 15% lower probability for an increase of energy expenditure of 1 kcal/kg/day [OR (CI) = 0.85 (0.78–0.92); *p* < 0.001].

As the RLS relationship to the pain/anxiety dimension of the EQ-5D questionnaire was of borderline significance, this association was also checked in the multivariate design. Variables selected as independent correlates of the pain/anxiety dimension of the EQ-5D were RRs [OR (CI) = 1.03 (1.01–1.05); *p* = 0.014], the GDS [OR (CI) = 1.30 (1.15–1.48); *p* < 0.001], and osteoarthritis [OR (CI) = 4.08 (1.93–8.70); *p* < 0.001].

## 4. Discussion

In the present study, we have assessed the prevalence of RLS in relation to the comprehensive geriatric assessment in two populations of older subjects, living in Poland and the United Kingdom. The obtained data shed some light on the epidemiology and possible pathophysiology of RLS in an advanced-aged population. We found that female sex, the number of medications taken per day and alcohol consumption are independent correlates of RLS in older adults. RLS together with chronic heart failure, osteoarthritis and urinary incontinence as well as physical activity level were independent correlates of the mobility dimension of the QoL. These data only partially conform to the results obtained in younger populations.

RLS can present alone or with comorbidities that make proper diagnosis difficult [34]. The background literature review covered correlates from various areas such as environmental factors, including heavy metals, dietary factors, lifestyle factors, medical conditions and drug interactions [5,12]. RLS has been associated with obesity [35], an increased body mass index and diabetes [36,37]. People with a normal weight had a lower risk of developing RLS [22]. Obesity increased the risk of sleep disturbances in the long term, and both obesity and sleep disturbances had negative effects on health [38]. Smoking was related to an increased RLS risk in several studies [22,35,36,39]. In our study, when multiple other potential covariates were assessed, the BMI or smoking status did not emerge as independent correlates of RLS presence.

Various comorbidities such as kidney disease, cardiovascular diseases, obstructive lung disease, diabetes mellitus, hypothyroidism, chronic liver disease, and neurological, rheumatological and respiratory disorders may accompany RLS [2,16,17,18,19,40]. In a cross-sectional study including 5324 subjects, high cholesterol and hypertension were associated with RLS [41]. In a large population-based study, having RLS at baseline was not a significant predictor of any subsequent cardiovascular risk factors and/or vascular diseases, but cardiovascular risk factors and diseases predicted the subsequent development of RLS in the general population [42]. Similarly, we have not found a correlation between vascular diseases and restless legs syndrome in our groups. Other age-related medical conditions associated with sleep disturbances, including respiratory diseases such as asthma, infections, digestive tract diseases, physical disability, dementia, pain, depression, anxiety, and sleep itself, were taken into account. Except for some tendency for depressive symptoms, no strong correlation of accompanying diseases was found in relation to RLS in our study.

Of special interest is a statistically significant and independent-of-other-factors correlation between the prevalence of RLS and the consumption of alcohol in older men and women, assessed separately, as well as a whole group from Poland and the United Kingdom. In several studies, an association between RLS and alcohol consumption has been suggested [35,36,43]. In an Indian population study, chronic daily alcohol consumption was found to be associated with RLS [44]. Aldrich and Shipley found that in a significant proportion of alcohol users, periodic leg movements contributed to sleep disturbance. Additionally, women who consumed two or more drinks per day were more likely to report symptoms of restless legs and to be diagnosed with RLS [45]. In contrast, a non-significant trend between a higher alcohol consumption and a lower risk of RLS was observed in one study [22]. Likewise, in a sample of 317 psychiatric inpatients, RLS was associated with lower alcohol consumption [37]. Interestingly, not indifferent to this could also be alcohol detoxification therapy. The results of the study by Jiménez-Jiménez et al. show that a significant percentage of patients undergoing alcohol detoxification therapy develop RLS symptoms [43]. Mackie et al. show that alcohol withdrawal may involve generalized physical and psychological discomfort and insomnia, and patients with alcohol withdrawal experience symptoms that meet the criteria for RLS [46].

Alcohol consumption, regardless of the dose, affects the occurrence of sleep disorders, including sleep onset latency, consolidation in the first sleep period and disruptions in the second half of sleep [47]. Alcohol adds to aging-related sleep disturbances and cognitive impairment by affecting the brain. This occurs because the function of neurons, neuron survival, cell migration and glia, and glial cell (astrocytes and oligodendrocytes) differentiation are disrupted by alcohol [47]. However, despite extensive inquiry, we could not find one study that confirms a statistical significance between alcohol usage and RLS in an older population, taking into account possible covariates. Therefore, our data strongly suggest an important contribution of alcohol consumption to the prevalence of RLS, independent of other multiple possible co-determinants.

We also found that the RLS group used significantly more medication than the group without RLS alongside the higher alcohol intake. It is possible that alcohol correlates with RLS directly as well as indirectly, e.g., by altering the effects of simultaneously taken medications. Drug–food/alcohol interactions are known to reduce the therapeutic effects of medications, as well as to induce potent adverse drug reactions [48]. Medications, such as antidepressants, antihistamines, and antipsychotics have been associated with RLS [1]. Secondary forms of RLS and possible interactions of medications require particular consideration in older adults [49]. Both alcohol and many medications are metabolized by the same enzymes in the liver, where pharmacokinetic interactions generally occur. Alcohol can interact with numerous classes of prescription medications including antibiotics, antidepressants, antihistamines, barbiturates, benzodiazepines, histamine H2 receptor antagonists, muscle relaxants, nonnarcotic pain medications and anti-inflammatory agents, opioids, and warfarin. It can also interact with over-the-counter medicines and herbal products, often with negative effects [50].

We were not able to demonstrate any relationship between physical activity and RLS. In one study, physical activity reduced the risk of RLS and had a positive effect on its symptoms [22]. The mechanism by which this happens remains unknown, although several theories have been postulated. For instance, better blood flow in the lower limbs or the increased release of endorphins and dopamine. Additionally, symptoms, which are experienced at rest are likely to be reduced in those who exercise [22]. In another study, exercise therapy significantly affected the manifestations of the illness. Stretching, fitness training, and reflexology were beneficial with no side effects [51].

In several studies RLS was associated with a significantly lower HRQL and a higher prevalence of depression [39,52]. In a few studies, in older adults, this was assessed in relation to some aspects of the CGA [53,54,55]. In a Turkish study of 492 subjects aged on average 73 years, sleep disturbance, depressive mood, the fear of falling, reduced QoL, frailty and polypharmacy were more prevalent in the RLS group [56]. In a recent small study (54 RLS patients, 30 people in the control group), RLS patients were prone to sleep disorders, anxiety, and depression. Sleep disorders increased with the severity of the RLS and had some influence on the patient’s cognitive function [53]. In a recent systematic review, a negative association between RLS and global cognition and attention was found. No significant differences in memory, executive function, or spatial cognition were observed between the RLS and control groups [54]. In a cross-sectional study of 1008 subjects aged ≥54 years, RLS did not predict incident disability for aggregate measures but was associated with an increased risk for specific limitations, including difficulty with climbing several stair flights, prolonged sitting, rising from a chair, stooping, moving heavy objects, carrying ten pounds, raising arms, or picking up a dime [57]. Interestingly, a large study on 90,337 Chinese adults showed that RLS was associated with increased incidents of perceived olfactory and taste dysfunction [58]. In a small study of 32 older patients, five with RLS, there was no association of RLS with clinical, laboratory or neurophysiological findings [55]. In the present study, we found a nonsignificant trend of greater depression in RLS sufferers. Additionally, we found that mobility difficulties were significantly more frequent in those with RLS which might suggest impairment related to the symptoms of RLS.

Several limitations of the present study should be acknowledged. Because the study was performed in selected populations, the results of this study may lack generalizability. The difference in alcohol consumption between the UK and Poland was high. Nevertheless, the participants both in Poland and the UK were questioned by the same investigator (MS). Therefore, this difference may be likely attributed to cultural dissimilarities between the two populations. To minimize potential biases, subjects without known anemia, chronic kidney disease, known iron or vitamin deficiency, terminal illness, major disabilities or cognitive impairment were included in the study. Nevertheless, the study lacks laboratory data on iron or vitamin deficiency. Although we have adjusted for the presence of major chronic diseases and CGA data, other factors might have influenced the occurrence of RLS. Possible biases in the interview (patients’ forgetfulness in older age) should also be taken into consideration. Therefore, current findings should be corroborated in future studies assessing other potential confounders in more general populations.

## 5. Conclusions

Female sex, the number of medications taken per day and alcohol consumption are independent correlates of RLS in older adults. RLS together with several chronic medical conditions and a low physical activity level were independent correlates of the mobility dimension of the QoL. Therefore, controlling alcohol consumption seems the most important clinical implication put forward to alleviate the burden of RLS in older adults, probably contributing also to a better QoL.

## Figures and Tables

**Table 1:** Characteristics for all subjects with and without restless legs syndrome (RLS).

	Subjects with RLS (n = 77)	Subjects without RLS (n = 169)	*p*-Value
Age (years)	80.0 ± 8.5 81 (74; 87)	78.6 ± 9.1 80 (72; 86)	0.26
Males (n; %)	13; 16.9	50; 29.6	**0.03**
Smoking (n; %)	3; 3.9	14; 8.3	0.21
Alcohol/7 days (n; %)	30; 39.0	27; 16.0	**<0.001**
Arterial hypertension (n; %)	56; 72.7	119; 70.4	0.71
Hypercholesterolemia (n; %)	29; 37.7	63; 37.3	0.95
Diabetes mellitus (n; %)	15; 19.5	41; 24.3	0.41
Myocardial infarction (n; %)	10; 13.0	24; 14.2	0.80
Ischemic heart disease (n; %)	27; 35.1	70; 41.4	0.34
Chronic heart failure (n; %)	36; 46.8	86; 50.9	0.55
Stroke (n; %)	8; 10.4	30; 17.8	0.14
Chronic obstructive pulmonary disease (n; %)	18; 23.4	30; 17.8	0.30
Osteoarthritis (n; %)	49; 63.6	88; 52.1	0.09
Osteoporosis (n; %)	27; 35.1	59; 34.9	0.98
Digestive tract diseases (n; %)	18; 23.4	33; 19.5	0.49
Past or present cancer (n; %)	8; 10.4	26; 15.4	0.29
Ophthalmologic diseases (n; %)	28; 36.4	54; 32.1	0.52
Depression (n; %)	17; 22.1	22; 13.1	0.07
Urinary incontinence (n; %)	27; 35.1	57; 33.9	0.86
Faecal incontinence (n; %)	12; 15.6	35; 20.8	0.33
Infections last year	1.0 ± 1.1 1 (0; 2)	1.1 ± 1.2 1 (0; 2)	0.49
Influenza vaccination last year (n; %)	41; 53.2	91; 54.5	0.86
Medications/day	7.0 ± 3.4 6 (5; 9)	5.9 ± 3.6 5 (3; 8)	**0.023**
RRs	143.0 ± 19.8 140 (130; 152)	143.2 ± 19.6 140 (130; 155)	0.94
RRd	80.1 ± 11.0 80 (75; 85)	81.6 ± 10.9 80 (75; 90)	0.33
BMI (kg/m^2^)	26.4 ± 4.8 26 (23; 30)	26.3 ± 4.2 26 (23; 29)	0.77
Calf circumference (cm)	34.9 ± 4.8 35 (32; 38)	34.6 ± 4.5 35 (32; 37)	0.62
ADL	4.9 ± 1.7 5.5 (5; 6)	4.6 ± 1.9 5.5 (4; 6)	0.19
IADL	5.3 ± 2.9 6 (3; 8)	4.4 ± 3.2 5 (1; 8)	**0.050**
TUG test (sec)	16.7 ± 9.5 14 (10; 21)	17.2 ± 10.7 14 (10; 21)	0.73
MNA	22.5 ± 4.2 24 (20; 26)	23.5 ± 3.7 24 (22; 27)	0.063
MMSE	24.8 ± 4.6 25 (21; 29)	23.8 ± 5.6 25(20; 29)	0.15
GDS	5.3 ± 3.7 5 (2; 8)	5.1 ± 3.7 5 (2; 8)	0.67
Energy expenditure kcal/kg/day	37.1 ± 4.3 35 (34; 39)	37.7 ± 5.0 36 (34; 40)	0.34
Stanford Moderate index	1.8 ± 1.5 2 (1; 3)	1.7 ± 1.6 1 (0; 3)	0.78

**Table 2:** QoL for all subjects with and without RLS examined using the EQ-5D questionnaire.

	Subjects with RLS (n = 77)	Subjects without RLS (n = 169)	*p*-Value
Mobility [%]			**0.01**
no problems	20.8	36.3	
moderate	63.6	44.0	
severe	15.6	19.6	
Self-care [%]			0.44
no problems	61.0	57.1	
moderate	26.0	23.2	
severe	13.0	19.6	
Usual activities [%]			0.31
no problems	37.7	42.9	
moderate	39.0	29.2	
severe	23.4	28.0	
Pain/discomfort [%]			0.10
no problems	11.7	23.2	
moderate	79.2	70.2	
severe	9.1	6.5	
Anxiety, depression [%]			0.25
no problems	29.9	36.9	
moderate	62.3	59.5	
severe	7.8	3.6	
Self-assessment of health (EQ-5D Visual Analogue Scale)	56.9 ± 18.9 50 (50; 70)	55.8 ± 20 50 (45; 70)	0.67

**Table 3:** Results of the multiple logistic regression with age, sex, country of participants, and all variables significantly related to RLS in bivariate associations.

Factor	Odds Ratios (95.0% Confidence Intervals)	*p*-Value
Age	1.008 (0.97–1.05)	0.69
Medications/day	1.11 (1.02–1.20)	0.018
IADL	1.04 (0.93–1.17)	0.51
Sex	3.22 (1.47–7.08)	0.0019
Country	1.07 (0.47–2.43)	0.87
Alcohol/7 days	5.04 (2.24–11.32)	<0.001

## Data Availability

Data will be available on reasonable request from the corresponding author.
